# Modeling spontaneous activity across an excitable epithelium: support for a coordination scenario of early neural evolution

**DOI:** 10.1186/1471-2202-16-S1-P120

**Published:** 2015-12-18

**Authors:** Oltman O de Wiljes, Ronald AJ van Elburg, Michael Biehl, Fred A Keijzer

**Affiliations:** 1 Department of Theoretical Philosophy, Groningen University, Groningen, the Netherlands; 2 Institute of Artificial Intelligence, Groningen University, Groningen, the Netherlands; 3 Johann Bernoulli Institute for Mathematics and Computer Science, Groningen University, Groningen, the Netherlands

## 

The reason why nervous systems first arose is an open question. Internal coordination models hold that nervous systems evolved initially as a device to coordinate internal activity, enabling multicellular effectors. They stress the use of multicellular contractility as an effector for motility: some sort of coordinative structure would have been necessary to have multicellular effectors in the first place. A recent example of such a model, the skin brain thesis, suggests that excitable epithelia using chemical signaling are a potential candidate as a nervous system precursor.

**Figure 1:**
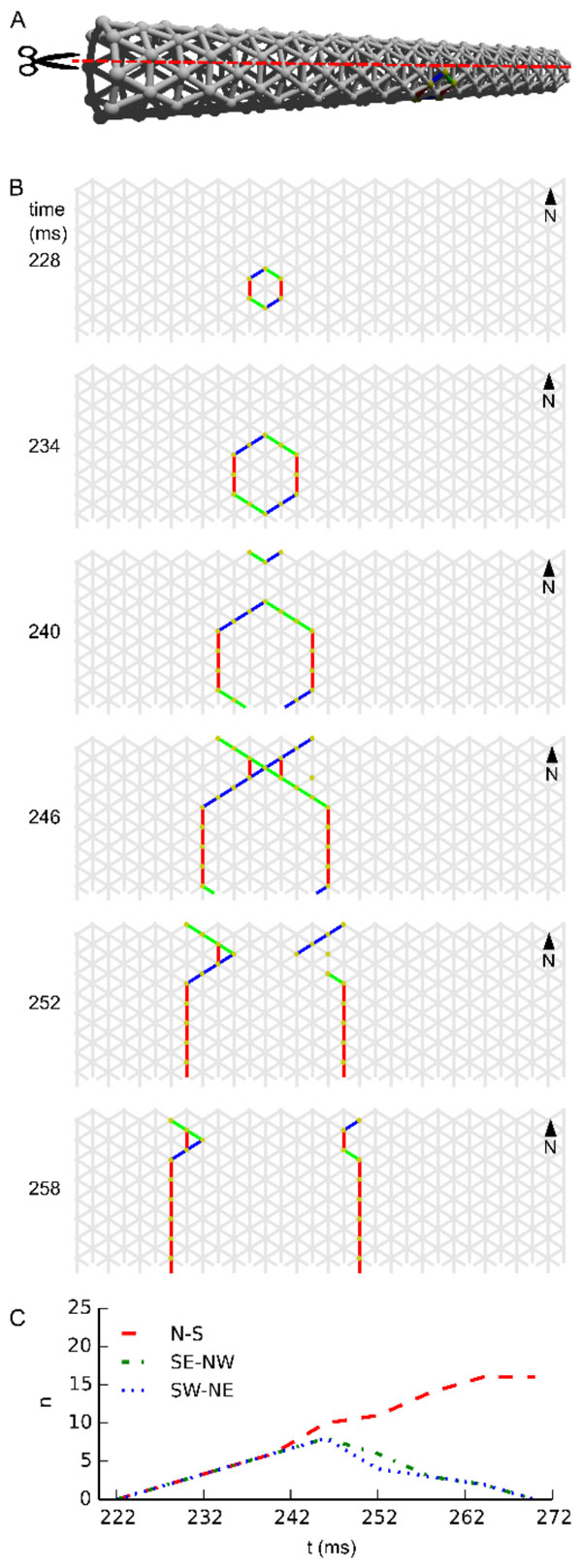
**Wave patterns on a long tube (length: 32 cells, circumference: 8 cells, noise rate: 0.1 Hz)**. **(A) **Network geometry: the scissors indicate the line at which the tube is cut for presentation in **B**. **(B) **Snapshots of network activity during 4 ms intervals in an illustrative phase of the dynamics. The wave fronts propagating transversely collide with each other, causing extinction due to the refractory period. Subsequently, the remaining wave-fronts propagating longitudinally dominate the dynamics and the North-South wave-front orientation dominates. **(C) **Temporal development of different wave-front orientations. Snapshot time markings are consistent with those in **B**.

We developed a computational model and a measure for whole body coordination to investigate the coordinative properties of such excitable epithelia. Using this measure we show that excitable epithelia can spontaneously exhibit body-scale patterns of activation (see Figure 1.). Relevant factors determining the extent of patterning are the noise level for exocytosis, relative body dimensions, and body size. In smaller bodies whole-body coordination emerges from cellular excitability and bidirectional excitatory transmission alone.

Our results show that basic internal coordination as proposed by the skin brain thesis could have arisen in this potential nervous system precursor, providing support that this configuration may have played a role as a proto-neural system and requires further investigation.

